# Catalysts for Change: The Role of Nonprofits in Solving Single Ventricle Heart Disease

**DOI:** 10.3390/jcdd9070220

**Published:** 2022-07-08

**Authors:** Kaitlin A. Davis, Diane M. Pickles, Kirstie E. Keller

**Affiliations:** Additional Ventures, Palo Alto, CA 94301, USA

**Keywords:** single ventricle, congenital heart disease, funding strategies, nonprofit, philanthropy

## Abstract

Single ventricle (SV) heart disease comprises a spectrum of complex congenital heart defects (CHDs), including hypoplastic left heart syndrome (HLHS), one of the most common causes of death amongst infants with CHD. Despite its incompletely defined etiology and a dearth of curative solutions, SV is a solvable problem that can be addressed by unifying a nascent field that is ripe for investment, in part due to its high economic impact and growth potential. Here, we explore the landscape of SV and identify areas of opportunity that will yield an outsized impact through strategic investment that focuses on synchronization across disciplines, community involvement, and infrastructure development, and argue that nonprofits are the appropriate catalyst to spark transformative innovation and impact in the form of functional cures.

## 1. Introduction

Single ventricle (SV) heart disease represents a series of complex conditions that are both life-threatening and life-altering, resulting in a significant economic burden on the healthcare and education systems, as well as patient and family communities. Such conditions include a myriad of diagnoses that are classified by anatomical or functional loss of one ventricle and necessitate invasive interventions and long-term care. Although the extensive impact of this disease is clear, the underlying etiology and quantifiable metrics on outcomes remain underexplored, and improvements in life-sustaining treatments and interventions underfunded. Traditional funding approaches often require large patient cohorts, a short-term horizon for returns, and a foundation of disease knowledge that allows for multiple targets to be tested, none of which hold true for SV, a field that is young, with a relatively small number of affected individuals. Thus, a new approach to funding methodology and deployment, particularly one guided by nonprofits, is critical to addressing the unmet needs of this community and to drive rapid progress towards curative solutions.

## 2. SV Incidence and Outcomes

Congenital heart defects (CHDs) are the most common birth defects affecting nearly 1 in 100 live births [1]. SV defects comprise almost 8% of all CHDs and include several disparate clinical diagnoses, including hypoplastic left heart syndrome (HLHS), the most common form of SV (2–3 per 10,000 live births), tricuspid atresia (1 per 10,000 live births), and pulmonary atresia with an intact ventricular system (PA-IVS; 4–5 per 100,000 live births) [2,3]. While SV defects were considered universally fatal only half a century ago, now most individuals born with SV undergo two to three live-saving, palliative surgeries in the first years of life, culminating in the Fontan operation [2,3]. Since the initial intervention described by Fontan and Baudet in 1971 [4], the type, number, and timing of surgeries undergone by SV patients has evolved considerably, drastically improving outcomes [3,5]. Indeed, at present, 80% of SV patients born today will live well into their 30s because of an improved care paradigm [5], as compared with the initial cohort of patients, whose post-surgery survival at 20 years was less than 50% [6]. Hence, these birth defects are no longer strictly pediatric illnesses; today, there are more than 1.4 million adults in the US living with CHDs—nearly 300,000 of these individuals have complex CHDs (cCHDs), such as SV [7].

As iterative improvements in clinical care, deepened understanding of related sequelae, and utilization of multidisciplinary approaches to patient care have improved outcomes, and knowledge in this area has grown, so too has the SV population; there were an estimated 70,000 Fontan patients living worldwide in 2017, and that number is projected to double in the next 20 years [5]. Life expectancy for these patients has increased significantly, and the goal has changed from simply surviving to thriving in life. Yet, in addition to the invasive surgeries required, most SV patients still experience profound co-morbidities and complications with shortened quality and length of life. Physical impacts are daunting and include circulatory failure, arrhythmia, liver fibrosis, and renal dysfunction; equally difficult for patients are the prevalent cognitive, neuropsychological, and behavioral deficits [5]. A normal longevity and quality of life are impossible, and the experience of living with an SV defect remains filled with physical and emotional pain and trauma.

## 3. Economic Burden of SV

While the burden of SV is objectively significant (Box 1), there is a shocking lack of data available on long-term outcomes and economic impact, with no comprehensive resource that describes the full spectrum. Therefore, efforts to describe the effects of this disease rely heavily upon data compiled on generalized CHDs to describe the effects of this disease, which fail to account for the specificity and extent of impacts unique to SV.

CHDs are among the costliest birth defects; though they comprise only 3.7% of hospitalizations for adolescents up to 20 years of age, they account for over 15% percent of total hospitalization costs for this age group, exceeding USD 6 billion annually [8,9]. In comparison with children who do not have CHDs, children with CHDs utilize 5 times more home health services, 8 times more medical equipment, 3 times more prescription medications, and 1.5 times more special education services than children who do not have CHDs [8]. The costs associated with cCHDs, such as SV, are even more staggering: 26.7% of all direct CHD-related medical costs are due to cCHDs, with the highest costs attributed to coarctation of the aorta and SV diagnoses, including HLHS and Tetralogy of Fallot [9].

The economic burdens extend beyond the healthcare system to the educational system and employment sector. Approximately 20–30% of all children with CHDs have at least one other physical problem or cognitive issue [9,10,11,12], significantly impacting quality of life, education, and future work opportunities and productivity. This number is significantly higher for individuals with complex cCHDs such as SV; children with cCHDs are as much as 50% more likely to require special education services than those without CHDs [13]. This population assumes a significant fraction of the total cost of special education services in the US, estimated at over USD 50 billion per academic year [14].

Perhaps most distressing are the economic impacts on patients and those who care for them. Adults with cCHDs are more likely than those with less severe forms of CHDs to have household incomes below USD 50,000 [13]; studies have found an employment rate of only 47% for adults with cCHDs [15]. The total burden of CHDs has been estimated at USD 500,000 per adult when considering medical costs and decreased earnings [16]; it is reasonable to infer that this burden would be significantly higher for cCHDs such as SV given the severity and complexity of the disease and of its care.

Parents and caregivers of children with rare diseases such as SV also experience many financial impacts from their child’s disease, including absenteeism from work, “presenteeism” (at work but unable to fully perform), forced retirement, and out-of-pocket healthcare costs not covered by insurance [17]. A survey of US households from 2011 to 2017 that included nearly 200 families living with CHDs found that nearly half of these families reported experiencing some level of financial hardship due to their child’s medical bills; 17% stated they could not pay their medical bills at all [18]. Financial burdens are more common among families with a child with CHDs than families who have children with other types of special health needs [8].

## 4. Funding Landscape

Federal investment in CHDs over the past five years has been disproportionately low when comparing per capita investment in other disease areas. For example, while the number of patients alive in the US with Alzheimer’s Disease and related dementias (ADRD) is 2.2 times greater than the number of patients alive with CHDs [7,19], federal funding in this category is 23 times greater (USD 3.2 billion invested in ADRDs compared with USD 138 million invested in CHDs in 2020) [20]. While CHDs are congenital diseases impacting individuals from birth, ADRD typically affects individuals over the age of 65; thus, when accounting for years of life, relative investment deviates even further.

Remarkably, the National Institutes of Health (NIH) did not recognize CHDs as a funding category prior to 2016, making data on prior investments difficult to accurately quantify [21], but also further underscoring the lack of emphasis on this field. Since its inception, federal funding in CHDs has remained stagnant, with a nominal increase of less than 5% annually, whereas NIH investment in Alzheimer’s has increased an average of 27% each year over the same period [20].

A small number of existing nonprofit foundations fund SV research, but these investments are difficult to quantify, in part because of their disparate distribution and siloed nature. To our knowledge, only one foundation has focused its biomedical research portfolio exclusively on SV [22]. Beyond this, philanthropic investment is relatively low from heart-focused organizations. While these contributions are not insignificant, there remains tremendous room to leverage and inflate these investment strategies to incite further impact and growth.

As a result, growth of the CHD field has been limited, and scientific progress has been impeded by a lack of critical resources and investigator support mechanisms. Additionally, CHDs represent dozens of conditions, further reducing the investment in any one specific defect and exposing that far too little research is focused specifically on SV, arguably the most complex, most deadly, and most expensive of all CHDs. Thus, continued efforts to identify novel treatments, improve medical care paradigms, and understand etiology and outcomes are critical, and urgently needed.

## 5. Research Landscape

The history of SV research is unique in that it is strongly tethered to advancements in surgical intervention, with the path towards a future for SV patients forged initially by pioneering surgeries. As patients have aged and reached adulthood, and additional challenges have emerged, the field expanded by necessity to include clinical care and translational research efforts. Now, a new era embracing basic science disciplines is emerging, as needed advances towards improved outcomes remain hindered by a limited understanding of the causes and biological mechanisms of SV and a lack of viable curative approaches [5]. However, the organic growth of the field is fragmented and siloed, signaling the need for an integrated approach across the various research stages to advance the treatment of SV.

Because of the nascency and complexity of this field, we conducted a landscape analysis to explore the next steps needed for advancements in research and development [23]. Here, we identified five key areas as critical for investment: developing foundational resources, understanding etiology, defining biological mechanisms of outcomes, developing evidence-based care strategies, and creating curative solutions. While this landscape analysis generated a robust roadmap for the community, the interdependencies of the key areas were not emphasized. In fact, there remains great need for integrated, combinatorial approaches, something that is underscored by the reality that investment in one type of research or discipline is insufficient to solve the complex challenges of SV, and that many of these problems cannot be solved without engaging multiple fields at one time. As such, strategic funding mechanisms that can assume the risk of early-stage research and provide additional layers of support are essential to progress.

## 6. SV: A Model for Innovative Investment

Unique characteristics of the SV field, including chronic underinvestment and urgent need for multidisciplinary collaboration, create an environment that would benefit from a more focused, hands-on approach; one that is poised for incredible societal impact. First, the nascency of the field, combined with the need for integrated approaches, emphasizes the opportunity to generate quick gains in knowledge that benefit a broad array of disciplines. Coupled with rapid growth and transformation in this age of technological advancements, particularly in basic science and genomics, there is ample opportunity to build a foundation on next level technology and cross-disciplinary collaboration. Second, the field has made significant advances despite a historical lack of funding, indicating that an infusion of capital could substantially increase the interest and outputs of the field, moving the needle far beyond incremental progress and building momentum that grows the field. Third, the development of needed infrastructure can build a platform for a multitude of research and trial endeavors. Investment in infrastructure decreases the barrier to entry for new investigators, easing collaboration and expansion beyond the bounds of a single field, and in particular, attracting industry partners. Lastly, the economic and healthcare burden are large, indicating a substantial return from a societal, and potentially, financial perspective. Though the SV field has been historically small, it is burgeoning and poised to deliver and emerge a leader—a model of innovation and impact for the families that need it most.

## 7. Nonprofits: Catalysts for Change

Nonprofit funders are uniquely positioned to accelerate progress in emerging fields, such as SV, as compared with other funding sources. While government and for-profit investment is often limited to financial support, nonprofits can offer creative models that strengthen the field while also developing potential assets. This hands-on method is critical, as the simple addition of more funding is not sufficient; the canonical “one research lab” approach cannot begin to solve a disease area such as SV, where there is multiplicity of presentation and outcomes, and a lack of understanding of biological and clinical mechanisms. Specifically, nonprofit organizations can act as a convener, invest in high-risk studies, and provide additional tools beyond capital, facets strengthened by a laser focus on a core mission.

First, as impact-focused entities, nonprofit organizations can act as a neutral convener, bringing together players across disciplines, sectors, and funding ethos. Such a position can generate collaborations, help source new talent, and identify areas of potential exploration, all mechanisms that allow for organic growth and novel ideas. As outlined above, SV can benefit from multi-disciplinary and team-based approaches; thus, nonprofits can yield an outsized impact by creating community amongst the SV field and adjacent fields.

Unlike more traditional funders, nonprofits can balance risk by spreading their investments across a broad portfolio, and one that is still focused on one primary mission [24]. For example, nonprofits can invest in very early basic science or in translational research, two areas often deemed too risky for government, industry, or academia [25], while coupling this investment with infrastructure development, more traditional grant mechanisms, and recurrent funding programs. In SV, nonprofits can seed investment in novel curative approaches, providing critical insight into which avenues to further support, develop crucial infrastructure as a platform for discovery, and create stable funding opportunities to solidify the field.

Finally, nonprofits can extend hands-on, holistic support combined with funding to bolster each investment and increase the likelihood of success. Challenges encountered in research are not limited to source or availability of funding, and often require a different type of investment. Such support can come in a variety of forms, such as external expertise, additional tools, or management, all requiring active engagement by the funder. In SV, providing resources to the community, such as a research roadmap, and support to burgeoning programs through matchmaking and operational support can help to build a strong foundation where investigators and organizations are positioned for success beyond the duration of an award.

## 8. Conclusions

SV is a solvable problem, but it will take courage and commitment from nonprofits to achieve curative solutions. Medical advancements have brought the SV patient population from a universally fatal diagnosis to the chance at reaching adulthood, but it will take the committed investment by nonprofits to achieve the curative solutions that this rare, yet highly impactful disease needs, and that patients and families deserve. The SV field is ready for investment, with a community poised to act and a roadmap for action, but it requires the right catalysts to come together in the nonprofit sector to create an environment that allows for idea testing, novel methods, and innovative thinking.

Box 1 **The impacts of single ventricle (SV) on patients and families.**

The impacts of single ventricle (SV) on patients and families:

- ***Significant co-morbidities:*** The Fontan circulation results in chronic elevation in venous pressure and decreased cardiac output which predisposes SV patients to many co-morbidities, including circulatory failure, ventricular dysfunction, atrioventricular valve regurgitation, arrhythmia, protein losing enteropathy, plastic bronchitis, liver fibrosis, and renal dysfunction [5].
- ***Neurodevelopmental disabilities*:** Although surgical outcomes have improved for those with cCHDs, neurological outcomes have not [26]. A review of studies around the neurodevelopmental outcomes of children with CHDs found that those with cCHDs were at significantly elevated risk for developmental delays in intelligence, academic achievement, language, visual construction and perception, attention, executive functioning, fine motor skills, gross motor skills, and psychosocial maladjustment [27]. Executive dysfunction increases with the severity of CHD [28].
- ***Economic burden on parents and caregivers:***  Financial burdens are more common among families with a child with CHD than families who have children with other types of special health needs [8]. A survey of US households from 2011 to 2017 that included nearly 200 families living with CHD found that nearly half of these families reported experiencing some level of financial hardship due to their child’s medical bills; 17% stated they could not pay their medical bills at all [18]
- ***Economic burden on adult patients:*** Studies have found an employment rate of only 47% for adults with cCHDs [15,29]. Adults with cCHDs are more likely than those with less severe forms of CHD to have household incomes below USD 50,000 [13]. The total burden of CHD has been estimated at USD 500,000 per adult when considering medical costs and decreased earnings [16].
